# Titania-Coated Silica Alone and Modified by Sodium Alginate as Sorbents for Heavy Metal Ions

**DOI:** 10.1186/s11671-018-2512-7

**Published:** 2018-04-12

**Authors:** D. Kołodyńska, M. Gęca, E. Skwarek, O. Goncharuk

**Affiliations:** 1 Department of Inorganic Chemistry, Faculty of Chemistry, Maria Curie Skłodowska University, M. Curie Skłodowska Sq. 2, 20-031 Lublin, Poland; 2 Department of Radiochemistry and Colloid Chemistry, Faculty of Chemistry, Maria Curie Skłodowska University, M. Curie Skłodowska Sq. 3, 20-031 Lublin, Poland; 3 Chuiko Institute of Surface Chemistry of National Academy of Sciences of Ukraine, 17 General Naumov Str, Kyiv, 03164 Ukraine

**Keywords:** Adsorption, Heavy metal species, Nanocomposite, Titania, Silica, Sodium alginate

## Abstract

The novel organic-inorganic biohybrid composite adsorbent was synthesized based on nanosized silica-titania modified with alginate within the development of effective adsorbent for heavy metal ions. Effects of metal species Cu(II), Zn(II), Cd(II), and Pb(II); concentrations; pH; temperature; and adsorption onto titania-coated silica (ST20) initial or modified by sodium alginate (ST20-ALG) were studied. The equilibrium and kinetic data of metal ions adsorption were analyzed using Langmuir and Freundlich adsorption models and kinetic models: pseudo first order, pseudo second order, intraparticle kinetic model, and Elovich. The maximum sorption capacities observed were higher for the ST20-ALG composite compared to the initial ST20 oxide for all studied metal ions, namely their values for ST20-ALG were 22.44 mg g^− 1^ for Cu(II) adsorption, 19.95 mg g^− 1^ for Zn(II), 18.85 mg g^− 1^ for Cd(II), and 32.49 mg g^− 1^ for Pb(II). Structure and properties of initial silica-titania ST20 and modified by sodium alginate ST20-ALG adsorbents were analyzed using nitrogen adsorption/desorption isotherms, ATR-FTIR, SEM-EDS, and pHpzc techniques.

## Background

Heavy metals species getting into the water from sewage and industrial wastes are one of serious threats for the environment. They are also dangerous for living organisms due to their toxicity and bioaccumulation. There are various methods to remove heavy metals species from aqueous media, e.g., ion exchange, chemical precipitation, membrane processes, and electrocoagulation. These techniques have many advantages, but in some cases, they are expensive and not very effective. Adsorption is the most common, efficient technique used for removal of heavy metal ions [1, 2]. Various sorbents are used for removal of contaminants from waters and wastewaters. For this aim, modified silica and silica-based systems with various functionalities could be used [3–7]. Fine silica particles have drawn much attention due to their surface structure, high specific surface area, low-cost production, and easy modification [8]. Fine titania particles are of great interest due to their unique properties and several potential technological applications such as pigments, photocatalysts, fillers and adsorbents, as well as their applications in solar cells and memory devices production [9]. Advantages of using titanium dioxide as an adsorbent are high surface area, high adsorption capacity, stability, non-toxicity, biological and chemical inertness, and high affinity for inorganic and organic pollutants [10–12]. In the paper by George et al. [13], TiO_2_ nanoparticles were successfully utilized for the removal of arsenic, antimony, lead, and cadmium in the presence of interfering ions from tap water. As it was reported in many papers, the nanostructured TiO_2_-based sorbents are characterized by improved photocatalytic properties toward inorganic and organic compounds due to the relatively high specific surface area, good mechanical stability, biocompatibility, and electrical properties [14]. However, when the suspension of nanosized TiO_2_ is used for adsorption of metal ions due to the fine grain size of such titania, aggregation of particles, loss of their activity, and difficulty with the recovery take place. These problems can be avoided by immobilizing nanosized TiO_2_ on various substrates, e.g., silica, by the sol-gel method.

Silica and titania composites can be obtained in the form of a blend, where both phases form individual particles with weak interactions, or as a mixture of titania and silica in the bulk and at a surface of carrier particles of one phase with tight contacts between both components. Upon surface modification of silica using alkoxysilane (in the presence of water), they form reactive organosilanols (RSi-OH) and alcohol is obtained as a by-product. Then the organosilanols undergo a condensation with the hydroxyl groups on the surface and inorganic oxide to give organofunctionals containing the Si–O–M covalent bonds. Composite microspheres silica with grafted nanosized TiO_2_ can exhibit novel properties that are not found in single oxides [15]. By combining the adsorption potential of silica with the photocatalytic properties of nanosized titania, it is possible to create integrated photocatalytic adsorbents with the improved photocatalytic potential. Numerous studies have reported synergistic effects in composites with nanosized TiO_2_ and SiO_2_ as well as active carbons, carbon nanotubes, and TiO_2_ [16–19]. According to the literature [20–23], the interaction between nanoscale silica and titania in composites leads to blue shifts in the range of binding energies of both O1s and Ti2p3/2 of silica-titania samples with respect to pure TiO_2_ are observed from X-ray photoelectron spectroscopy (XPS), due to the formation of Ti-O-Si bonds because of Ti^4+^ cations intrusion into tetrahedral sites of the silica lattice. The formation of Ti-O-Si bonds leads to greater electronegativity of Si to that of Ti, therefore the effective positive charge on Ti rises and the effective negative charge on O drops.

Alginate, cellulose, and chitosan are biopolymers which can be used as carriers for controlled drug release, membranes with regulated permeability, sensor devices, and artificial muscles [24, 25]. Their sorption ability toward heavy metal ions was also proved as for individual biopolymers [25] as well as in composites [26]. Therefore, the alginate modification of nanosized TiO_2_-SiO_2_ composite is a very simple and cheap method preventing aggregation of their particles and improving sorption properties. Sodium alginate (ALG) consists of sequences of M (M-blocks) and G (G-blocks) residues to form MG sequences (MG-blocks) [24]. Chelation of metal cations, such as Ca(II), by carboxylate groups of MG-blocks causes the cross-linking of alginates. This type of sorbents is not well-known and some basic rules of kinetics and adsorption of heavy metal ions especially when accompanied by the photoreduction from aqueous solutions have not been fully understood. In the case of organic-inorganic composites, oxide particles can improve thermal properties, self-sustaining capability to work under different conditions, better hydrophobic interactions with polymers, and specific properties such as chemical binding capability for a variety of compounds.

The titania-coated silica microspheres functionalized with potassium ferrocyanide and impregnated in calcium alginate were used for efficient removal of cesium from the aquatic media [27]. It was found that the maximum sorption of cesium was attained in the pH range 7.5–8.5 and the equilibrium time 24 h. The maximum sorption capacity of the beads was 23.55 mg/g and the sorption followed the Langmuir isotherm. The removal of arsenic using alginic acid was investigated by Mina and Hering [28]. The optimal durability and efficiency of arsenic removal was achieved at pH 4.0. This yield increased with the increasing iron content. At the initial As(V) concentration 400 μg L^− 1^ and pH 4.0 after 120 h, As(V) removal rate was equal to 94%. In the paper by Fulazzaky et al. [29], it was proved that due to the valence electrons associated with the O–H functional groups of titania PVA-alginate beads, Cd(II) ions can be precipitated in the form of Cd(0). The alginate-TiO_2_ sorbent was also used for adsorption and removal of cationic (Methylene Blue, MB) and anionic (Methyl Orange, MO) dyes from waters and wastewaters [30]. The obtained beads exhibited a strongly enhanced adsorption of MB compare to the nanopowder samples (55 vs. 6.5%).

The aim of this work was the synthesis of organic-inorganic composite by modification of titania-silica oxide with alginate and the comparison of the adsorption properties of initial silica-titania and it composite with alginate with respect to the heavy metal ions. Analysis and determination of the regularities of adsorption of bivalent metal ions such as Cu(II), Zn(II), Cd(II), and Pb(II) using approach of the adsorption kinetics, adsorption capacity, the analysis of adsorption mechanisms, and their relationship with the structure of the adsorbent were the priorities of this study for development of effective sorbents for heavy metals adsorption from aqueous solutions.

## Methods

### Materials

Fumed silica A-50 (pilot plant of the Chuiko Institute of Surface Chemistry, Kalush, Ukraine, specific surface area S = 50 m^2^ g^− 1^) was used as the initial material. Titanium isopropoxide Ti[OCH(CH_3_)_2_]_4_ (TTIP) (Sigma Aldrich, 98%) dissolved in 2-propanol (Sigma Aldrich) was used as a titania precursor. Sodium alginate (ROTH) and calcium chloride hexahydrate CaCl_2_⋅6H_2_O (CHEMPUR) were used to prepare ST20-ALG beads.

### Composites Synthesis

Silica A-50 (with added 2-propanol at 40 °C and stirred to form a fine dispersion) was modified by addition of TTIP solution in 2-propanol heated at 200 °С for 2 h, then cooled to room temperature in air to add water providing TTIP hydrolysis. Then the mixture was heated to 80 °С to enable to form amorphous titania. Then it was heated at 110 °С to remove the solvent. The residue was calcined at 800 °С in air for 1 h. All operations were carried out in a reactor equipped with a PTFE stirrer and air purge system. The final material with the A-50 silica matrix and grafted nanosized titania was labeled as ST20.

The oxide sorbent was modified by applying sodium alginate solutions. The appropriate amount of ST20 was mixed with 1% solution of sodium alginate. Then the mixture was added dropwise using a peristaltic pump (type PP1 B-05A, Zalimp) to a 2% solution of CaCl_2_ at flow rate 2.5 cm ^3^ min^− 1^. The beads were left in CaCl_2_ solution for 24 h. Then they were washed several times with distilled water. The prepared composite sorbent was labeled as ST20-ALG.

### Fourier Transform Infrared Spectroscopy

In order to characterize ST20 and ST20-ALG before and after Cu(II) and Pb(II) sorption, Fourier transform infrared (FTIR) spectroscopy with a Cary 630 (Agilent Technologies) using an attenuated total reflectance mode (ATR-FTIR) was applied. The analysis was performed in the range of 4000–400 cm^− 1^.

### Nitrogen Adsorption-Desorption Measurements

Nitrogen adsorption-desorption measurements were performed at 77.35 K using a Micromeritic ASAP 2020 adsorption analyzer with ultra-high purity nitrogen. All samples were outgassed under vacuum at 110 °C for 2 h prior to the measurements. The specific surface area (*S*_BET_) was calculated according to the standard BET method [31]. The total pore volume *V*_p_ was evaluated from the nitrogen adsorption at *p*/*p*_0_ ≈ 0.98–0.99, where *p* and *p*_0_ denote the equilibrium and saturation pressure of nitrogen at 77.4 K, respectively [32].

The nitrogen desorption data were used to compute the pore size distributions (PSD, differential *f*_V_(*R*) ~ d*V*_p_/d*R* and *f*_S_(*R*) ~ d*S*/d*R*) using a self-consistent regularization (SCR) procedure under non-negativity condition (*f*_V_(*R*) ≥ 0 at any pore radius *R*) at a fixed regularization parameter *α* = 0.01. A complex pore model was applied with cylindrical (C) pores and voids (V) between spherical NPNP packed in random aggregates (CV/SCR method) [33].

### Scanning Electron Microscopic

Scanning electron microscopic (SEM) images with energy dispersive spectroscopy (EDS) were recorded by means of a Quanta 3D FEG (FEI) apparatus. TEM micrographs were recorded using a JEM100CX II apparatus.

### Adsorption Studies

Batch experiments were conducted at room temperature using 0.1 g of a sorbent added into an Erlenmeyer flask with 20 cm^3^ solutions containing Cu(II), Zn(II), Cd(II), and Pb(II) ions at concentrations in the range of 50–250 mg L^− 1^. The samples were shaken on a mechanical shaker (Elpin Plus 357 type, Poland) from 1 to 240 min (amplitude 7, 180 rpm). The concentrations of metal ions were measured by means of a Spectr AA 240 FS (Varian) atomic absorption spectrometer. The sorption of metal ions (mg/g) was calculated according to a standard procedure.

In order to investigate the effect of adsorbent amount, 0.05, 0.1, and 0.15 g of ST20 or ST20-ALG were used per 20 cm^3^ solution of metal species. The initial concentration of Cu(II), Zn(II), Cd(II), and Pb(II) was 100 mg L^− 1^. The temperature effect on Cu(II) sorption on ST20 and ST20-ALG was studied at 20, 40, and 60 °C. The initial concentration of Cu(II) was 100 mg L^− 1^ and the amount of adsorbent was 0.1 g/20 cm^3^ (5 g L^− 1^).

The adsorption percentage was calculated based on the difference between the amounts of Cu(II), Zn(II), Cd(II), and Pb(II) ions in the initial solution and after the sorption process. The effect of the phase contact time was studied based on Cu(II), Zn(II), Cd(II), and Pb(II) sorption on ST20 and ST20-ALG. The initial concentration of each metal ion was 100 mg L^− 1^ and the amount of adsorbent was 0.1 g/20 cm^3^. The effect of different initial concentrations of Cu(II) (50–250 mg L^− 1^) on sorption using ST20 and ST20-ALG was examined. The solution pH was measured using a pH meter PHM82.

In this study, various diffusion and kinetic models (pseudo first order [34], pseudo second order [35, 36], intraparticle diffusion [37], and Elovich [38, 39] models) were used in order to determine the contact time required to reach the equilibrium and to understand the rate of the sorption process. Knowledge on the process rate provides useful information about the influence of metal species adsorption on ST20 and ST20-ALG.

The pseudo first order model described by the equation below assumes that the rate of adsorption is proportional to the number of free sites, unoccupied by heavy metals or other impurities.

1 $$ \mathit{\ln}\left({q}_e-{q}_t\right)=\mathit{\ln}{q}_e-{k}_1t $$

where *q*_t_ is the amount of heavy metal ions adsorbed at time *t* (mg g^− 1^), *q*_e_ is the amount of heavy metal ions adsorbed at equilibrium (mg g^− 1^), and *k*_1_ is the pseudo first order model constant (L min^− 1^).

The pseudo second equation is presented below:

2 $$ \frac{t}{q_t}=\frac{1}{k_2{q}_e^2}+\frac{t}{q_e} $$

where *k*_2_ is the pseudo second order model constant (g mg^− 1^ min^− 1^).

The intraparticle diffusion equation is as follows:

3 $$ {q}_t={k}_i{t}^{1/2}+C $$

where *k*_i_ is the intraparticle diffusion model constant (mg g^− 1^ min^-1/2^) and *C* is the diffusion constant (mg g^− 1^).

The Elovich model is used to confirm the chemisorption process:

4 $$ {q}_t=\frac{1}{b}\mathit{\ln}(ab)+\frac{1}{b}\mathit{\ln}(t) $$

where *a* is the initial sorption (mg g^− 1^·min^− 1^) and *b* is related to the extent of surface coverage and activation energy for chemisorption (desorption constant) (g mg^− 1^).

### Determination of Point of Zero Charge, pH_PZC_

The drift method and the titration method were used for pH_PZC_ determination. To determine pH_PZC_, a dispersion of 0.5 g ST-20 sample in 100 cm^3^ of a 0.01 M NaCl solution previously adjusted to a predetermined pH in the range of 1 to 14 was shaken for 1 day until an equilibrium pH was reached. Then the pH of each solution was measured. The difference between the initial (pH_i_) and at equilibrium (pH_e_) pH values was plotted vs. pH_i_.

## Results and Discussion

### Adsorbent Characterization

The textural characteristics of ST20 and ST-ALG were determined using the nitrogen adsorption/desorption isotherms.

**Fig. 1:**
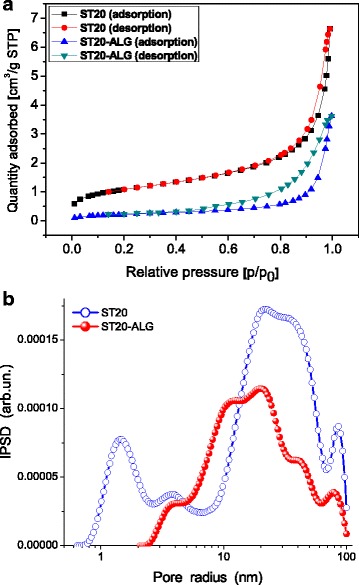
**a** Nitrogen adsorption–desorption isotherms at 77 K and **b** incremental pore size distributions for ST-20 and ST20-ALG

It was found that the S_BET_ surface area of ST20 was equal to 53 m^2^ g^− 1^ (Table 1) than is close to the value of S_BET_ of A-50 (52 m^2^ g^− 1^). Figure 1 shows the nitrogen adsorption-desorption isotherms for ST-20 and ST20-ALG and the pore size distributions (PSDs) obtained from the nitrogen adsorption isotherms. The PSD curves for ST20 and ST20-ALG differ due to polymer filling of voids between oxide nanoparticles.

**Table 1:** The physicochemical parameters of ST20 and ST20-ALG

Parameter	ST20	ST20-ALG
Particle size range	15–20 nm	1.2 mm beads or powder
Moisture content	> 5%	> 30%
S_BET_ (m^2^ g^− 1^)	53	42
*V*_total_ (cm^3^ g^− 1^)	0.205	0.178
*S*_macro_ (m^2^ g^− 1^)	13	11
*V*_macro_ (cm^3^ g^− 1^)	0.148	0.128
*S*_meso_ (m^2^ g^− 1^)	40	32
*V*_meso_ (cm^3^ g^− 1^)	0.057	0.032

It was found that titania grafted onto silica A-50 is characterized by much lower crystallinity than titania synthesized alone because of silica inhibitory effects [40]. Modification of ST20 by sodium alginate can change diffusion of adsorbate into pores (voids) and provide a possibility for further surface modification. Therefore, sodium alginate solution was used here. On the other hand, mechanical weakness of alginates and relatively poor adhesion should also be mentioned.

**Fig. 2:**
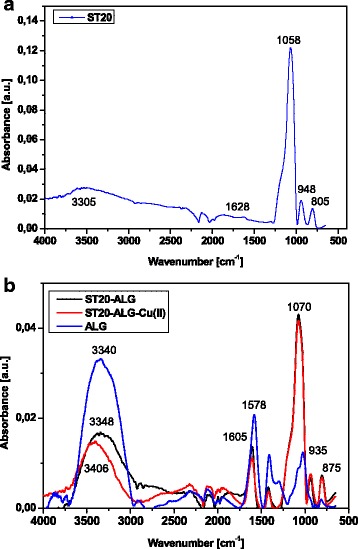
FTIR-ATR spectra of ST20 **a** before and **b** after ALG modification and Cu(II) ions sorption

The FTIR technique, especially with ATR mode, is one of the most effective tools for characterization of surface functionalities at a silica surface, such as Si–OH [41]. Isolated Si–OH groups result in a strong sharp band at 3750 cm^− 1^, while that of free silanol (Si–OH) in the organosilicon compounds appears at about 3690 cm^− 1^ with a sharp band. In the ATR-FTIR spectra, the ≡SiOH groups and adsorbed water give a broad band of ν_OH_ at 3605 cm^− 1^ (Fig. 2).

The bands at 1058 cm^− 1^ for ST-20 and 1070 for ST20-ALG and 805 cm^− 1^ are attributed to silica. The band at 1067 and 805 cm^− 1^ indicates asymmetric and symmetric O-Si stretching vibrations [42]. The band at 935 cm^− 1^ corresponds to Si-O-Ti vibrations, for which the characteristic range is 928–952 cm^− 1^. After the sorption of Cu(II) and Pb(II) on ST20, changes in the spectra are observed. The alkyl groups have been removed by calcination and it is evident due to the absence of the C-H peaks in the FTIR spectra.

**Fig. 3:**
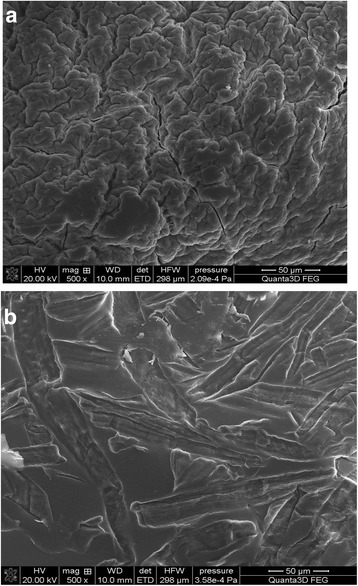
SEM-EDX analysis of ST20-ALG and ST20-ALG-Cu: scale, magnification (mag), voltage (HV) and vacuum pressure are shown in panel

The ATR-FTIR spectra are consistent with the XRD and TEM results [40] revealing that the particulate morphology of titania correspond to crystalline anatase. In the case of ST20, the titania particles ranged between 15 and 20 nm. When ST20 was modified by ALG, the beads were also characterized by a spherical shape and average diameter varying from 0.5 mm to about 2 mm. From SEM images (Fig. 3), it can be seen that a film covering the ST20 surface of ‘brain pattern’ is formed and the porous surface is visible. After the Cu(II) adsorption, the surface was covered by thin flakes.

### pH Effect

**Fig. 4:**
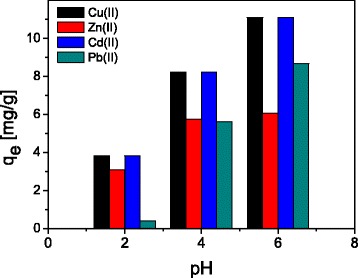
pH effect on the sorption of Cu(II), Zn(II), Cd(II), and Pb(II) on ST20-ALG

The pH value plays an important role with respect to the adsorption of different ions on oxide surfaces. In order to determine the effect of pH, the pH values of sample solutions were adjusted to a range of 2–6. The obtained results presented in Fig. 4 indicates increasing adsorption of all studied metal ions with the increase pH values form 2 to 6 on ST20-ALG composite.

The result of the effect of pH on the adsorption of Cu(II), Zn(II), Cd(II), and Pb(II) revealed that the quantitative adsorption (>  95%) is found for Pb(II) and Cd(II) in the pH range 5–6 and therefore the initial pH (without adjustments which was 5.5 for Cu(II), 5.0 for Zn(II), 5.0 for Cd(II), and 5.0 for Pb(II)) was taken as the compromise condition.

Also determination of point of zero charge, pH_PZC_ is very important in order to understand the adsorption mechanism. The pH_PZC_ is the pH value at which the solid surface in electrolyte solution takes neither positive nor negative charge. In the solutions with the pH value lower than the point of zero charge, the adsorbent surface is positively charged and for pH values greater than the point of zero charge, the surface is negatively charged. It is well-known that cation adsorption occurs at pH greater than pH_PZC_, while anion is favored at a pH lower than pH_PZC_. It was found that pH_PZC_ of ST20 was 7.8 and ST20-ALG 8.2. It should be mentioned that the pH_PZC_ of the anatase powders is 6.2.

For TS20, it was also found that below the pH_PZC_, that adsorption occurs by ion exchange mechanism:

5 $$ 2\left(\equiv \mathrm{Si}-\mathrm{OH}\right)+{\mathrm{M}}^{2+}\rightleftarrows 2\left(\equiv \mathrm{Si}\mathrm{O}\right)\mathrm{M}+{2\mathrm{H}}^{+} $$

6 $$ \equiv \mathrm{Si}-\mathrm{OH}+{\mathrm{MOH}}^{+}\rightleftarrows \equiv \mathrm{Si}\mathrm{OMOH}+{\mathrm{H}}^{+} $$

and above the pH_ZPC_ by bonding:

7 $$ 2\left(\equiv \mathrm{Si}-\mathrm{OH}\right)+\mathrm{M}{\left(\mathrm{OH}\right)}_2\rightleftarrows {\left(\equiv \mathrm{Si}\mathrm{OH}\right)}_2\mathrm{M}{\left(\mathrm{OH}\right)}_2 $$

### Kinetic of Adsorption

**Fig. 5:**
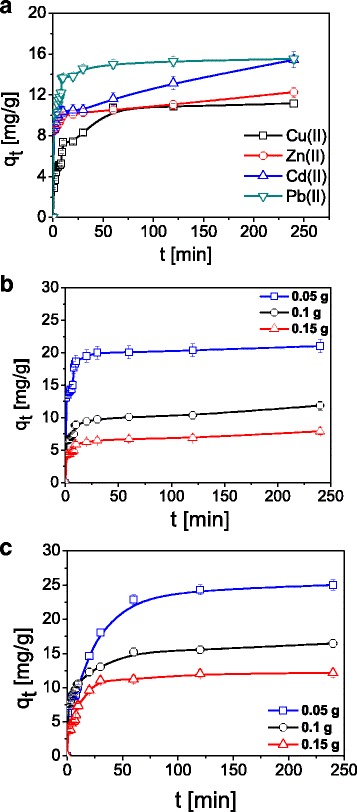
Comparison of **a** the amount of Cu(II), Zn(II), Cd(II), and Pb(II) ions sorbed on ST20 depending on the time and **b**, **c** adsorbent amount effect of Cu(II) ions sorption on **b** ST20 and **c** ST20-ALG (C_0_ = 100 mg L^− 1^, *m* = 0.1 g, *t* = 1–240 min, pH_Cu_ = 5.45, pH_Zn_ = 5.01, pH_Cd_ = 5.37, pH_Pb_ = 5.24, *T* = 293 K, *A* = 7, rpm 180)

The effect of time phase contact on Cu(II), Zn(II), Cd(II), and Pb(II) sorption on ST20 is presented in Fig. 5. With the increasing contact time, the adsorption capacity as well as sorption percentage (%S) increase initially and then reach the equilibrium. After 60 min, adsorption of Cu(II) and Zn(II) ions reaches 80% and then achieves plateau of 99% after 240 min. The quick sorption of Pb(II) on ST20 can suggest domination by the chemical sorption.

**Fig. 6:**
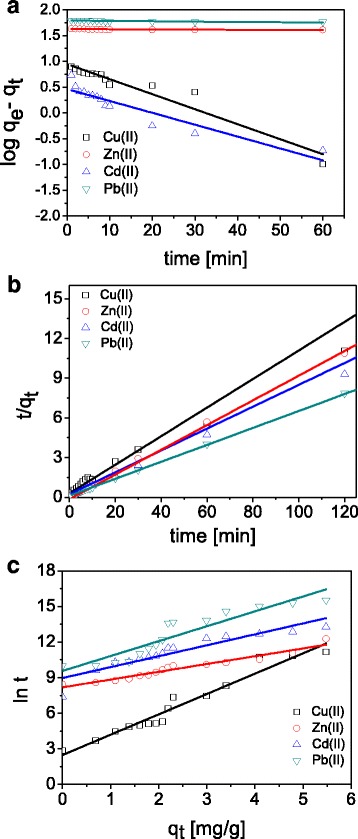
Kinetic plots for Cu(II), Zn(II), Cd(II), and Pb(II) ions sorption on ST20 **a** pseudo first order model, **b** pseudo second order model, and **c** Elovich model

The kinetics of Cu(II), Zn(II), Cd(II), and Pb(II) on ST20 was determined using pseudo first order, pseudo second order, intraparticle diffusion, and Elovich models as described above. The parameters of kinetic models for Cu(II), Zn(II), Cd(II), and Pb(II) adsorption on ST20 are listed in Table 2. The analogous results for ST20-ALG are presented in Table 3. The fitting results are also given in Fig. 6. The pseudo first order model is suitable only in the case the adsorption process occurs very rapidly and therefore it did not gave desirable results in our investigations. The pseudo second order kinetic model for each metal ion on ST20 affords the best fitting (*R*^2^ > 0.999). This indicates that the rate controlling step in the adsorption process is the chemisorption one.

**Table 2:** Kinetic parameters for Cu(II), Zn(II), Cd(II), and Pb(II) ions sorption on ST20

Model	Parameter	Cu(II)	Zn(II)	Cd(II)	Pb(II)
PFO	*q*_1,cal_	8.85	7.28	4.91	8.84
	*k*_1_	0.067	0.041	0.053	0.032
	*t*_1/2_	0.008	0.012	0.018	0.019
	*R* ^2^	0.9321	0.9970	0.8691	0.8219
PSO	*q*_2,cal_	9.26	12.10	10.71	15.62
	*k*_2_	0.039	0.030	0.060	0.034
	*h*	3.365	4.334	6.860	8.406
	*t*_1/2_	0.034	0.024	0.016	0.022
	*R* ^2^	0.9993	0.9990	0.9995	0.9999
IPD	*k*_i1_ (mg g^−1^ min^−1/2^)	1.29	1.67	1.65	1.27
	*C*_1_	4.92	7.66	6.74	8.30
	*R* ^2^_1_	0.9008	0.9614	0.9790	0.8754
	*k*_i2_ (mg g^−1^ min^−1/2^)	0. 82	0.20	0.26	0.35
	*C*_2_	8.14	9.54	10.86	12.51
	*R* ^2^_2_	0.9337	0.9179	0.9348	0.8393
	*k*_i3_ (mg g^−1^ min^−1/2^)	0.23	0.13	0.08	0.09
	*C*_3_	7.94	9.02	12.06	14.20
	*R* ^2^_3_	0.9983	0.9696	0.9937	0.9204
EM	*a*	17.23	10.24	18.65	18.64
	*b*	0.57	1.47	1.30	0.77
	*R* ^2^	0.9494	0.9437	0.9311	0.8629

*PFO* pseudo first order, *PSO* pseudo second order, *IPD* intraparticle diffusion model, *EM* Elovich model

**Table 3:** Kinetic parameters for Cu(II), Zn(II), Cd(II), and Pb(II) ions sorption on ST20-ALG

Model	Parameter	Cu(II)	Zn(II)	Cd(II)	Pb(II)
PFO	*q*_1,cal_	6.29	7.21	6.23	8.38
	*k*_1_	0.022	0.003	0.028	0.001
	*t*_1/2_	0.04	0.008	0.007	0.009
	*R* ^2^	0.9532	0.8573	0.9686	0.9387
PSO	*q*_2,cal_	10.19	11.47	12.75	19.99
	*k*_2_	0.020	0.013	0.034	0.063
	*h*	2.040	1.691	3.957	2.200
	*t*_1/2_	0.002	0.0011	0.003	0.0032
	*R* ^2^	0.9913	0.9986	0.9984	1.0000
IPD	*k*_i1_ (mg g^−1^ min^−1/2^)	0.94	1.29	0.64	1.28
	*C*_1_	3.25	1.92	5.84	3.95
	*R* ^2^_1_	0.9532	0.9008	0.9399	0.9642
	*k*_i2_ (mg g^−1^ min^−1/2^)	0.62	0.59	0.44	0.64
	*C*_2_	4.27	5.02	6.65	16.25
	*R* ^2^_2_	0.9142	0.7714	0.9489	0.8927
	*k*_i3_ (mg g^−1^ min^−1/2^)	0.54	0.23	0.49	0.03
	*C*_3_	4.67	7.94	6.12	19.50
	*R* ^2^_3_	0.9566	0.5984	0.9692	0.8214
EM	*a*	8.12	7.45	5.97	9.79
	*b*	0.59	0.57	0.75	0.64
	*R* ^2^	0.9516	0.9494	0.9123	0.8688

*PFO* pseudo first order, *PSO* pseudo second order, *IPD* intraparticle diffusion model, *EM* Elovich model

In the case of the intraparticle diffusion model based on the plot of *q*_t_ vs. *t*^1/2^, the multi linearity with three adsorption stages can be distinguished (data not presented). It is commonly known that when the plot passes through the origin, only the intraparticle diffusion is the rate limiting step. The results show that the plots do not pass through the origin which points out that the adsorption process is not the only a rate controlling step. The first step is associated with metal ion diffusion from the solution to the ST20 external surface or the boundary layer diffusion of the solute molecules. In the case of Cd(II) or Pb(II), the first step is fast. The second stage indicates that the metal ions got into the pores of ST20 due to the intraparticle diffusion. The third stage is affected by the diffusion into the small pores. The intraparticle diffusion comes to the end because the maximum adsorption is reached. Moreover, it was found that the obtained straight lines do not pass through the origin and also the correlation coefficients are lower than these for PFO and PSO, therefore this model is not appropriate to explain the nature of studied process.

As for the Elovich model, the parameters (*1*/*b*) and (*1*/*b*)ln(*ab*) obtained from the slope and intercept of the linear plot of *q*_t_ vs. ln t are also listed in Tables 2 and 3. The value of *1*/*b* indicates the number of sites accessible to adsorption while (*1*/*b*)ln(*ab*) is the adsorption quantity when ln*t* is equal to zero. This value promotes understanding the adsorption behavior. The correlation coefficients for all metal ions are 0.8926–0.9494 for ST20 and 0.8688–0.9516 for ST20-ALG, which indicates unsuitability of this model for metal ions adsorption on ST20.

### Adsorption Isotherms

The most commonly applied isotherm models are still the Langmuir and Freundlich ones. The Langmuir model (LM) is based on the assumptions of homogeneous adsorption sites and absence of interactions between the adsorbed components. The non-linear form of the Langmuir equation is:

8 $$ {q}_e=\frac{q_0{K}_L{c}_e}{1+{K}_L{c}_e} $$

where *q*_0_ is the maximum adsorption capacity (mg g^− 1^) and *K*_L_ is the energy of adsorption (L/mg).

The Freundlich model (FM) is empirical assuming the heterogeneous adsorbent surface and exponentially increasing adsorption capacity of the adsorbate:

9 $$ {q}_e={K}_F{c}_e^{1/n} $$

where *K*_*F*_ is the adsorption capacity characteristic of the Freundlich model (mg g^− 1^) and *1*/*n* is the Freundlich constant connected with the surface heterogeneity.

The parameters *K*_F_ and *n* were calculated based on the linear relationship *logc*_*e*_ vs. *logq*_*e*_. Moreover, *1*/*n* values indicate the type of isotherm which can be irreversible (1/*n* = 0), favorable (0 < 1/*n* < 1), and unfavorable (1/*n* > 1).

Additionally, the Dubinin-Radushkevich (D-RM) isotherm model was chosen to establish the adsorption mechanism of Cu(II), Zn(II), Cd(II), and Pb(II) ions sorption on ST20 and ST20-ALG. It can be used to describe adsorption on both homogeneous and heterogeneous surfaces:

10 $$ {q}_e={K}_{DR}{\exp}^{-{\beta \varepsilon}^2} $$

where *q*_e_ is the theoretical isotherm capacity (mg g^− 1^), *K*_DR_ is the constant related to the mean free energy of adsorption per mole of the adsorbate (mol^2^ J^− 2^), and *ε* is the Polanyi potential. The Polanyi potential can be expressed as follows:

11 $$ \varepsilon = RTln\left(1+\frac{1}{c_e}\right) $$

where *R* is the gas constant (8.314 J mol^− 1^ K^− 1^), *T* is the temperature (K), and *c*_e_ is the concentrations at equilibrium (mg L^− 1^) [36–38].

**Fig. 7:**
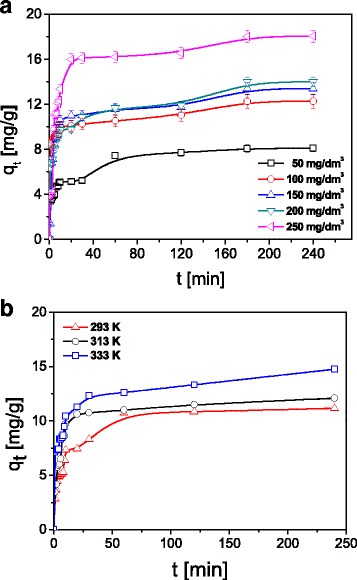
Comparison of the amount of sorbed Cu(II) ions depending on **a** concentration on ST20 and **b** temperature on ST20 (C_0_ = 50, 100, 150, 200, 250 mg/L, *m* = 0.1 g, *t* = 1–180 min, pH_Cu_ = 5.45, *T* = 293 K, *A* = 7, rpm 180)

In the first stage of the investigations, it was proved that the amount of the metal ions sorbed on ST20 and ST20-ALG increases with the increasing initial concentrations. The exemplary results and the effect of the initial concentration for Cu(II) ions sorption on ST20 are presented in Fig. 7.

The equilibrium isotherms of Cu(II), Zn(II), Cd(II), and Pb(II) ions on the investigated ST20 and ST20-ALG sorbents were obtained by examining the relationship of *c*_*e*_/*q*_*e*_ vs. *c*_*e*_ in the case of LM, log*q*_*e*_ vs. log*c*_*e*_ in the case of FM, and ln*q*_e_ vs. *ε*^2^ in the case of D-RM*.* The model fitting results are shown in Table 4.

**Table 4:** Adsorption parameters for Cu(II), Zn(II), Cd(II), and Pb(II) ions sorption on ST20 and ST20-ALG obtained from LM, FM, and D-RM

Isotherm	Parameters	Cu(II)	Zn(II)	Cd(II)	Pb(II)
ST20					
LM	*q*_e_ (mg g^−1^)	20.26	17.63	16.73	26.89
	*K*_L_ (L mg^− 1^)	0.056	0.065	0.049	0.184
	*R* ^2^	0.9999	0.9998	0.9992	0.9999
FM	*K*_F_ (mg g^−1^)	4.12	6.25	2.73	6.82
	*n*	3.266	5.301	3.431	4.609
	*R* ^2^	0.9657	0.9563	0.9388	0.9551
D-RM	*K*_DR_ (mg g^−1^)	0.001	0.011	0.001	0.002
	*E* (kJ mol^−1^)	11.794	13.009	11.408	13.077
	*R* ^2^	0.9825	0.9847	0.9900	0.8021
ST20-ALG					
LM	*q*_e_ (mg g^−1^)	22.44	19.95	18.85	32.49
	*K*_L_ ((L mg^−1^)	0.027	0.044	0.123	0.227
	*R* ^2^	1.0000	0.9999	0.9999	0.9999
FM	*K*_F_ (mg g^−1^)	4.12	5.12	3.33	9.54
	*n*	3.113	4.224	2.551	3.789
	*R* ^2^	0.9612	0.9222	0.9439	0.9472
D-RM	*K*_DR_ (mg g^−1^)	0.002	0.002	0.001	0.001
	*E* (kJ mol^−1^)	11.247	12.778	11.333	15.651
	*R* ^2^	0.9001	0.9873	0.9355	0.9982

*LM* Langmuir isotherm model, *FM* Freundlich isotherm model, *D-RM* Dubinin–Radushkevich isotherm model

**Fig. 8:**
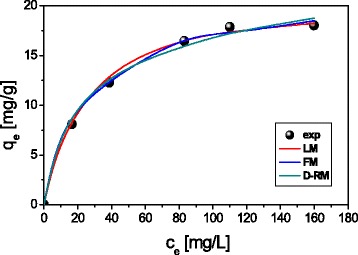
The Langmuir (LM), Freundlich (FM), and Dubinin-Radushkevich (D-RM) isotherms of Cu(II) on ST20 (C_0_ = 50–250 mg L^− 1^, *m* = 0.1 g, *t* = 180 min, pH_Cu_ = 5.45, *T* = 293 K, *A* = 7, rpm 180)

The Langmuir isotherm model gave the highest correlation coefficient values, showing that the adsorption of heavy metal ions on ST20 and ST20-ALG was described better by this model (Fig. 8). Thus formation of the monolayer can be more presumable than heterogeneous surface sorption. Additionally, the Langmuir isotherm assumes uniform energies of adsorption on the surface and the absence of interactions among the adsorbed molecules.

The maximum sorption capacities for Cu(II) 22.44 mg g^− 1^, for Zn(II) 19.95 mg g^− 1^, for Cd(II) 18.85 mg g^− 1^, and for Pb(II) 32.49 mg g^− 1^ were obtained at an initial metal concentration in the range 50–250 mg L^− 1^, pH 5, ST20-ALG dose 1 g/L, and the phase contact time 240 min. For ST20 sorbent, they were a bit lower and equal to 20.26, 17.63, 16.73, and 26.89 mg g^− 1^, respectively. Conversion of the maximum sorption capacities values into mmol g^− 1^ allows comparison of the number of adsorbed cations Cu(II), Zn(II), Cd(II), and Pb(II). These values show that the amount of adsorbed metal ions decreases in a sequence corresponding to an increase in their atomic weights: _63_Cu (0.35 mmol g^− 1^for ST20-ALG and 0.31 mmol g^− 1^ST20) > _65_Zn (0.31 mmol g^− 1^ for ST20-ALG and 0.27 mmol g^− 1^ for ST20) > _112_Cd (0.17 mmol g^− 1^for ST20-ALG and 0.15 mmol g^− 1^ ST20) > _207_Pb (0.16 mmol g^− 1^ for ST20-ALG and 0.13 mmol g^− 1^ for ST20). Such a sequence contradicts the regularities of ions adsorption by their position in the lyotropic series (the Hoffmeister series) implying the formation of a hydrated shell around the cations inversely depends on their radii and, accordingly, the adsorption of ions of the same valence should increase as their radii increase because of the hydration shell decrease and an ion polarity increase. The violation of this regularity can be explained by the higher affinity of such ions as Cu(II) and Zn(II) to the surface of adsorbents at very close atomic radii sizes.

### Coexisting Anions Effect

**Fig. 9:**
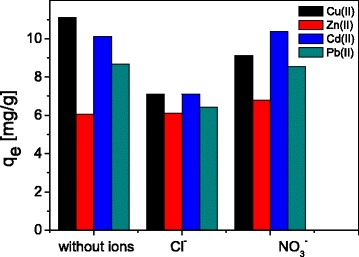
Coexisting ions effect on the sorption of Cu(II), Zn(II), Cd(II), and Pb(II) on ST20-ALG

When studying the phenomena of cations adsorption, it is necessary to take into account the electrolyte composition of the solution, since coexisting anions depending on their species can either promote cation adsorption or reduce it. Such influence significantly contributes to the study of the pattern of adsorption of cations, therefore the effects of coexisting ions Cl^−^ and NO_3_^−^ at the concentration of 100 mg L^− 1^ on the adsorption of Cu(II), Zn(II), Cd(II), and Pb(II) on ST20-ALG were also investigated. In these experiments, the solutions of 100 mg L^− 1^ Cu(II) containing the added interfering ions were shaken with ST20-ALG for 240 min. It can be seen that when adsorbing various cations, the coexisting Cl^−^ and NO_3_^−^ anions have different effects (Fig. 9).

Thus, in the case of the Zn(II) adsorption, the addition of Cl^−^ and NO_3_^−^ does not change the maximum adsorption value. During Cu(II) adsorption, the presence of both of anions decreases adsorption capacity, although in varying degrees: the effect of NO_3_^−^ is relatively minor, but influence of Cl^−^ is very noticeable. The effect of NO_3_^−^ on adsorption of Cd(II) and Pb(II) is absent, whereas in the presence of Cl^−^, the adsorption capacity decreases noticeably. Such regularities show the complexity of the cations adsorption process on the surface of adsorbents, and the need for taking into account such factors as the presence and concentration of indifferent and non-indifferent electrolytes affecting processes by both changing the surface charge of adsorbents and the structure of the double electric layer formation, as well as influence on the diffusion process.

## Conclusions

The successful application of nanosized TiO_2_-based sorbents such as ST20 and ST20-ALG for heavy metal ions removal from waters and wastewaters was proved. In this study, the adsorption of heavy metal ions such as Cu(II), Zn(II), Cd(II), and Pb(II) on ST20 and ST20-ALG sorbents was studied. Additionally, the modification of oxide silica-titania composite with alginate as a very simple and cheap method for prevention of aggregation of nanosized TiO_2_-SiO_2_ particles was confirmed. ST20 modification is a rapid method for intensification of its adsorption properties. Cu(II), Zn(II), Cd(II), and Pb(II) ions sorption is predominantly affected by a combination of factors, namely the initial metal concentration, pH, sorbent dosage, and the phase contact time. The study indicates that the heavy metal ions such as Cu(II), Zn(II), Cd(II), and Pb(II) exhibit the high affinity for ST20-ALG as well as for ST20.

The Langmuir isotherm model gave the highest correlation coefficient values, showing that during the heavy metal ions adsorption on ST20 and ST20-ALG, the monolayer formation is more presumable than heterogeneous surface sorption. The observed equilibrium values of maximum adsorption of all studied metal ions are higher for the organo-inorganic ST20-ALG composite than for the ST20 oxide.

The study of adsorption kinetics has shown that with the increasing contact time, the adsorption increase dramatically in the first 10 min, reaches 80% in 60 min, and then reaches the equilibrium plateau of 99% in 240 min. Comparison of different models for the interpretation of kinetic adsorption data has shown that the most adequate model for both types of adsorbents (inorganic ST20 and organo-inorganic ST20-ALG) is the pseudo second order kinetic model for each metal ion on ST20 affords the best fitting (*R*^2^ > 0.9990). This indicates that the rate controlling step in the adsorption process is the chemisorption one. In addition, the analysis of kinetic data using the intraparticle diffusion model showed the effect not only of the adsorption process itself but also diffusion of metal ions from solution to the outer surface and penetration into the pores of the adsorbent on the adsorption rate.

The effects of coexisting ions Cl^−^ and NO_3_^−^ are different for the sorption of the studied metal ions. Thus, Сu(II) adsorption decreases in the presence of both of coexisting ions Cl^−^ and NO_3_^−^, NO_3_^−^ ions does not have any effect on sorption of Cd(II) and Pb(II), while the presence of Cl^−^ ions reduces adsorption, and the effect of coexisting ions is absent for Zn(II) sorption.

## Abbreviations

ATR: Attenuated total reflectance
D-RM: Dubinin–Radushkevich isotherm model
EM: Elovich kinetic model
FM: Freundlich isotherm model
FTIR: Infrared spectroscopy
IPD: Intraparticle diffusion model
LM: Langmuir isotherm model
PFO: Pseudo first order model
PSO: Pseudo second order
*S*_BET_: Specific surface area
SEM: Scanning electron microscopy
